# Population-based structural variation discovery with Hydra-Multi

**DOI:** 10.1093/bioinformatics/btu771

**Published:** 2014-12-02

**Authors:** Michael R. Lindberg, Ira M. Hall, Aaron R. Quinlan

**Affiliations:** ^1^Department of Biochemistry and Molecular Genetics, ^2^Center for Public Health Genomics, University of Virginia, Charlottesville, VA, USA, ^3^Department of Medicine, ^4^The Genome Institute, Washington University School of Medicine, St. Louis MO, USA and ^5^Department of Public Health Sciences, University of Virginia, Charlottesville, VA, USA

## Abstract

**Summary:** Current strategies for SNP and INDEL discovery incorporate sequence alignments from multiple individuals to maximize sensitivity and specificity. It is widely accepted that this approach also improves structural variant (SV) detection. However, multisample SV analysis has been stymied by the fundamental difficulties of SV calling, e.g. library insert size variability, SV alignment signal integration and detecting long-range genomic rearrangements involving disjoint loci. Extant tools suffer from poor scalability, which limits the number of genomes that can be co-analyzed and complicates analysis workflows. We have developed an approach that enables multisample SV analysis in hundreds to thousands of human genomes using commodity hardware. Here, we describe *Hydra-Multi* and measure its accuracy, speed and scalability using publicly available datasets provided by The 1000 Genomes Project and by The Cancer Genome Atlas (TCGA).

**Availability and implementation:** *Hydra-Multi* is written in C++ and is freely available at https://github.com/arq5x/Hydra.

**Contact:** aaronquinlan@gmail.com or ihall@genome.wustl.edu

**Supplementary information:** Supplementary data are available at *Bioinformatics* online.

## 1 Introduction

We present an extension of *Hydra* (Quinlan *et al.*, 2010), our structural variant (SV) discovery software that, like many extant tools, was designed to detect SV in a single genome using discordant paired-end alignment signals. *Hydra-Multi* generalizes the *Hydra* algorithm to multiple samples/libraries and extends its scalability to incorporate information from many genomes simultaneously. Variant discovery from multiple samples has been a staple of SNP and INDEL discovery (Koboldt *et al*., 2009; Larson *et al.*, 2011; Lee *et al.*, 2010; McKenna *et al.*, 2010), and has been shown to provide substantial improvement in accuracy over the single-genome strategy. Therefore, it is logical to make use of all available data in SV detection, especially due to the ever-increasing number of datasets from large projects such as The Cancer Genome Atlas (TCGA) and The 1000 Genomes Project (1KGP). We previously applied multisample SV discovery in a study of genome instability in mouse-induced pluripotent stem cell lines (Quinlan *et al*., 2011). However, the algorithm employed for that study was limited to a handful of samples. *Hydra-Multi* was therefore developed to call SVs in a large number of ‘tumor-normal’ pairs (Malhotra *et al.*, 2013). In previous studies, the standard cancer genome workflow consisted of calling SVs in a tumor and a matched normal and subsequently compared the calls in each sample to find putative somatic mutations. Such ‘tumor-normal’ comparisons are fraught with somatic misclassifications (i.e. predicting that a variant is somatic when it is actually in the germline) where evidence of SV is found in the tumor but not the normal. This problem is exacerbated when shallow coverage is obtained for normal samples, leading to a greater number of false somatic SV predictions in the tumor sample. In contrast, directly integrating multiple datasets can prevent somatic misclassification in cases where the supporting alignments do not exist in the matched normal but do exist in the genomes of other normal samples.

Other algorithms (Handsaker *et al.*, 2011; Hormozdiari *et al.*, 2011) have employed similar strategies; however, these frameworks either scale poorly on commodity hardware or simply perform *post hoc* genotyping rather than directly combining all data during SV discovery. Genotyping after discovery can suffer from instances where ample coverage may not be present, and therefore SV breakpoints are missed in the discovery phase.

## 2 Methods

*Hydra-Multi’s* read-pair clustering strategy is similar to that of *Hydra* and a detailed description of both the workflow (Supplementary Fig. S1) and algorithm can be found in the Supplementary Materials. Fundamentally, *Hydra-Multi* differs from *Hydra* by accounting for the intra- and inter-sample differences in the size and variance of fragments observed among DNA libraries, thus enabling *Hydra-Multi* to infer which read-pairs from different samples corroborate the same SV despite variability in the absolute mapping distances. The algorithm extracts discordant read-pairs from each sample in parallel (one process per input bam file) and then segregates them by the chromosome and alignment orientation observed on each end. This process isolates the distinct sets of alignments that have the potential to support each rearrangement class (e.g. deletions, inversions, etc.) on a chromosome (or pair of chromosomes). Each chromosome/orientation set is then sorted by their left-most chromosomal coordinate using a memory-efficient k-way merge-sort algorithm. This allows for population-scale SV discovery under the memory constraints of typical commodity computing hardware. Sorting discordant alignments by chromosome coordinate allows the discovery algorithm to ‘sweep’ across chromosomes in search of clusters of discordant alignments that support a common SV breakpoint. Because discordant alignments are presegregated by chromosome and orientation pairs, clusters can be identified in parallel. We use a greedy algorithm to integrate the supporting alignments into a single breakpoint call. A cluster is terminated by a mapping whose start coordinate is to the ‘right’ of the current cluster’s rightmost end coordinate; such a mapping cannot support the same breakpoint as the mappings already in the cluster. A cluster may also be terminated in regions of aberrantly high read-depth. These regions typically reflect poorly assembled regions of the reference genome and can cause numerous false positives and excessive runtimes. Such loci can be avoided by limiting the number of discordant alignments that may be attributed to a putative cluster, as informed by the depth of the input datasets. All alignments from all samples are examined together and, by tracking the sample or library from which each supporting alignment originated, the algorithm accounts for the expected variance in fragment size for each sample when screening for supporting alignments in a given SV cluster. As such, the final output of *Hydra-Multi* contains the number of supporting alignments observed in each sample for every SV breakpoint call, thereby allowing analyses of the presence of SV breakpoints in each sample.

## 3 Results

### 3.1 Accuracy

**Fig. 1.**
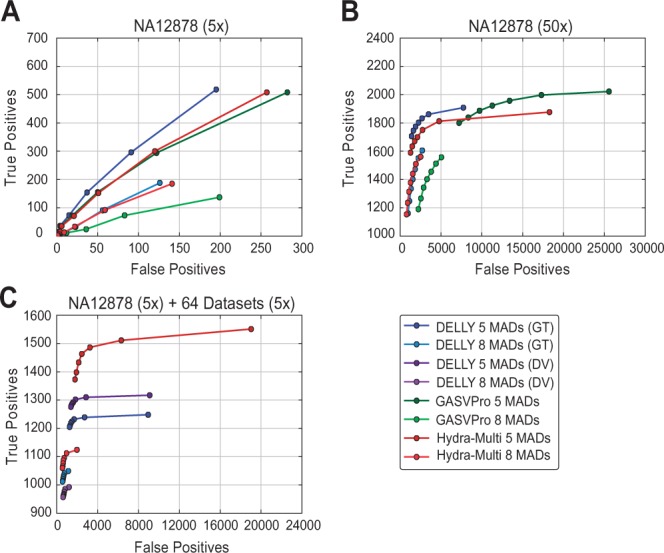
Receiver operating characteristic curves describing deletion detection in NA12878 from three scenarios. The relative accuracy of *Hydra-Multi* (red) was compared with both *DELLY* (blue and purple) and *GASVPro* (green) in three analyses that each compared fragment size parameters of 5 and 8 median absolute deviations (MADs) (Supplementary Methods). Each plot displays the relationship between the number of true and false positives at varying levels of minimum alignment support (4–10 read-pairs). A true positive was defined as detection of one of the 3077 non-overlapping truth set deletions where both intervals from a predicted deletion breakpoint intersected with both of the truth set deletion breakpoint intervals. In order to make a fair comparison across all tools, each predicted breakpoint was represented as two 200 bp intervals that faithfully represent the region implicated by the original SV call. A list of regions to exclude based on excessively high read-depth were used on both the truth set and putative call sets (Supplementary Methods). The three situations used to assess the three tools are as follows: **(A)** The 50× NA12878 dataset was subsampled to 5× and analyzed. **(B)** The 50× NA12878 data were analyzed. **(C)** The subsampled 5× NA12878 dataset was analyzed concurrently with 64 randomly selected datasets of ∼5× coverage from 1KGP. Total support was evaluated as the total number of read-pairs across all datasets analyzed. The presence of a deletion in NA12878 by *DELLY* was inferred by both the reported genotype (GT) and by observing at least one high-quality variant pair (DV) in NA12878. Only GT was reported in the single dataset analyses, as GT and DV are functionally the same when requiring 4–10 read pairs of support. In both single and joint analyses using *Hydra-Multi*, the contribution of at least one read pair by NA12878 was required. *Note*: *GASVPro* does not simultaneously run on multiple datasets

To evaluate the relative accuracy of the predictions made by *Hydra-Multi*, we compared it with two widely used SV discovery tools, *GASVPro* (Sindi *et al.*, 2009) and *DELLY* (Rausch *et al.*, 2012). We chose to compare with these methods because they have been shown to outperform various other methods and they have been employed in the analysis of large-scale datasets from TCGA and 1KGP. However, we note that multisample variant calling is a relatively new and as yet unpublished feature of *DELLY*, and that to our knowledge *GASVPro* is not capable of multi-sample calling. We measured each tool’s ability to detect deletions by analyzing NA12878 from the 1KGP CEPH population in three typical scenarios (Fig. 1). The lack of a reliable truth set for hundreds to thousands of samples limited the size and scope of the performance analysis to a trusted set of 3077 validated, non-overlapping deletions in NA12878 (Mills *et al.*, 2011).

Our analysis revealed that *DELLY* has the best performance in terms of sensitivity and specificity when a single dataset is analyzed in isolation (Fig. 1A and B), and that *Hydra-Multi* has the best performance when 65 datasets are subjected to joint multisample analysis (Fig. 1C). The slightly superior performance of *DELLY* on a single dataset is not surprising given that it utilizes both paired-end and split-read alignment signals during SV discovery, whereas *Hydra-Multi* and *GASVPro* rely solely on paired-end alignments. Hydra-Multi was explicitly designed for joint analysis of a large number of datasets, and in this usage scenario it exhibits significantly improved sensitivity; however, *Hydra-Multi* also exhibits competitive performance in single dataset usage scenarios, outperforming *GASVPro* and achieving near parity with *DELLY* in most cases. In the single dataset analyses (Fig. 1A and B), we found that the true positive rates were fairly consistent amongst the different tools, with the main difference being the number of false positives. Although all methods have a high false positive rate under minimum evidence parameters required to achieve high sensitivity, the false positive ranges under different parameters suggests that this can be largely ameliorated by parameter tuning and filtering. This exemplifies the well-recognized difficulty of performing sensitive and accurate SV detection from short-read sequencing data, although we note that the 1KGP truth set is known to be incomplete and therefore the number of false positives we report here is likely to be an upper bound.

The advantage of multisample analysis is apparent by the dramatic improvement in SV detection sensitivity for both *Hydra-Multi* and *DELLY* when the 5× NA12878 dataset is co-analyzed with 64 additional 5× genomes (Fig. 1C) relative to when the 5× NA12878 dataset is analyzed by itself (Fig. 1A). In this comparison, *Hydra-Multi* has substantially higher sensitivity than *DELLY* with a tolerable increase in the number of false positives at a given evidence threshold. Taken together, these results show that *Hydra-Multi* is competitive with other best-in-class SV detection tools in terms of sensitivity and specificity when run on a single dataset in isolation, and that *Hydra-Multi* excels at joint multi-sample SV calling.

*Hydra-Multi* was originally developed to explore the mechanisms driving complex genomic rearrangements among 129 whole genome sequencing datasets (64 tumors and 65 matched normal tissues, Supplementary Table S1) from TCGA (Malhotra *et al.,* 2013). After filtering, we obtained a final set of 33 218 high-confidence SV breakpoints among the 129 genomes. As expected, >80% (27 039) of these breakpoints were observed in matched normal samples and inferred to be germline variants. Because each tumor-normal pair originated from the same individual, we expect that a comparison of the genetic distance between the 129 genomes will reveal this relationship. To test this, we applied hierarchical clustering to 11 944 high-quality germline deletion and duplication calls (≤1 Mb). For each germline SV, the presence or absence of the breakpoint was measured in the 129 samples. As expected, each tumor-normal pair is most closely related to one another (Supplementary Fig. S2).

**Fig. 2.**
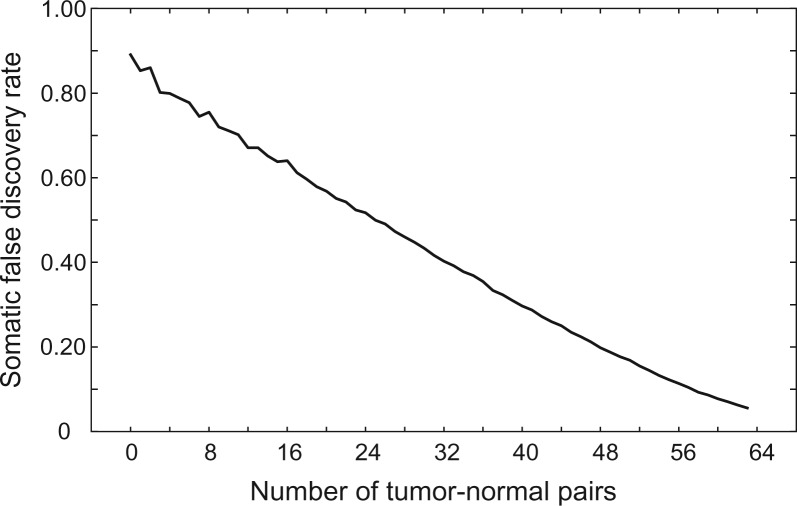
Reduction in the somatic SV FDR for tumor-specific mutations by simultaneously integrating data from 128 TCGA samples. The somatic FDR is the predicted rate at which somatic SV breakpoints are false, either due to false positive SV calls or due to inherited germline SVs that have been misclassified as somatic due to false negatives. For this experiment, we identify false somatic calls by their presence in a single normal genome but not in the paired tumor genome or any of N additional tumor-normal pairs (X-axis)

The remaining 6502 SVs found in Malhotra *et al.* (2013) were ‘private’ SVs observed in only one of the 129 samples. As expected given that solid tumor genomes are often highly rearranged, over 95% (6179) of the private breakpoints were found in a single tumor genome. In contrast, a mere 323 (5%) of the breakpoints were observed in the genome of a single matched normal DNA sample. By assuming that all variants private to a normal genome are false and that the absolute number of false positive somatic calls is similar between tumor and normal datasets, we inferred the somatic false discovery rate (FDR) to be 5.2% (323/6179) (Malhotra *et al.,* 2013). This may be an overestimate given that a fraction of the variants private to a single normal sample are likely to be real, owing to occasional loss of heterozygosity in the matched tumor. We further note that approximately half of the apparent false positives are small deletion variants that are likely misclassified due to varied resolution amongst samples caused by differing insert size distributions. Here, we emphasize that the fact that at most 5.2% of the somatic rearrangement predictions are incorrect is the direct result of integrating data from all 129 tumor and normal genomes. In contrast, were we to predict somatic SVs using the common practice of solely comparing each tumor to its matched normal, 89.1% of the predictions would have been false using the somatic FDR estimation approach described above (Malhotra *et al.,* 2013). Alternatively, were we to utilize a *post hoc* somatic SV classification strategy based on integrating results after performing single-sample variant calling separately on all 129 genomes, 21.9% of somatic SV calls would have been incorrect (versus 5.2% for joint calling). These results further underscore the superiority of joint variant calling for somatic SV detection. Indeed, as illustrated in Figure 2, the somatic FDR decreases dramatically as additional tumor-normal pairs are used for discovery, arguing that large cancer genomics studies will greatly minimize spurious somatic calls by adopting this multi-sample SV detection strategy.

The main effect of joint variant calling appears to be increased sensitivity, thus minimizing the misclassification of germline SVs as somatic SVs due to false negatives. However, it may also be true that some fraction of false positive SV calls arise systematically in multiple samples and are classified as ‘germline’ variants, thus further reducing the somatic FDR.

### 3.2 Speed and scalability

The main motivation for the development of *Hydra-Multi* was fast runtime and scalable performance, and in these terms it greatly outperforms the other tools. Under the same usage scenarios as presented in Figure 1, *Hydra-Multi* was 2–13× (2.2, 2.3 and 12.5×) faster than *DELLY* and 12–14× (12.8 and 13.9×) faster than *GASVPro*, and required merely 3.2 h to analyze the set of 65 5× datasets (Table 1), whereas DELLY required 39.9 h. *Hydra-Multi* achieved these fast runtimes while using substantially less memory than the other tools: e.g. in the 65 dataset comparison (Table 1), *Hydra-Multi* used merely 1.9 Gb of memory while *DELLY* used 41.3 Gb, which represents a 22-fold difference. Importantly, *Hydra-Multi’s* performance allows for a much larger number of datasets to be co-analyzed on a single machine, which improves variant detection sensitivity and simplifies data processing workflows for large-scale studies. A large 500-dataset scenario was simulated using repeated inputs of the 5× NA12878 dataset, revealing tractable runtime (∼30 h) and memory usage (6.9 Gb) for *Hydra-Multi* on a single commodity server with 128 Gb of RAM. In contrast, it takes *DELLY* more than 2 weeks and >70 Gb of RAM to analyze 500 NA12878 datasets (Table 1).

**Table 1.** Memory usage and runtime performance from four scenarios

	Hydra-Multi		DELLY		GASVPro	
	Maximum memory	Total runtime	Maximum memory	Total runtime	Maximum memory	Total runtime
NA12878 (5×)	1.9 Gb	17 min	1.6 Gb	37 min	1.1 Gb	217 min
NA12878 (50×)	1.8 Gb	145 min	7.1 Gb	337 min	7.8 Gb	2017 min
NA12878 (5×) + 64 Datasets (5×)	1.9 Gb	192 min	41.3 Gb	2 392 min	N/A	N/A
500 NA12878 (5×)	6.9 Gb	1817 min	70.7 Gb	21 258 min	N/A	N/A

The relative speed and scalability of *Hydra-Multi* was compared with the other tools by measuring the maximum memory used per process and runtime with Runit (https://github.com/lh3/misc/tree/master/sys/runit). *Hydra-Multi* (8 processors) and *DELLY* were parallelized (32 threads). *GASVPro* ran as a single process/thread, never exceeding the Java Virtual Machine allocation of 20 Gb. From top, we analyzed the following datasets: a 5× NA12878 dataset obtained by subsampling the 50× NA12878 dataset; the 50× NA12878 dataset; the 5× NA12878 dataset combined with 64 additional ∼5× datasets from 1KGP; 500 copies of the 5× NA12878 dataset. *Note*: *GASVPro* cannot jointly analyze multiple datasets (indicated by ‘N/A’).

*Hydra-Multi’s* low memory usage is achieved primarily through the use of a memory assisted, k-way merge sorting algorithm and its speed is achieved largely through parallelization of both the discordant extraction and assembly phases (Supplementary Fig. S1). Extraction and assembly are coarsely parallelized, i.e. one processor per dataset and chromosome/orientation set, respectively. Under recommended parameters, discordant read-pair extraction predominates algorithm runtime and scales linearly with the amount of input data when supplied a single processor (Supplementary Fig. S3A). Supporting this assertion, there is a direct relationship between the number of discordant read-pairs and runtime (Supplementary Fig. S3B). By parallelizing the work, the cost of examining additional data is reduced. Both the disk-based sort and parallelization make scalability a central strength of *Hydra-Multi,* thus enabling incorporation of an extremely large number of datasets for SV discovery.

Given the increasing number of large-scale genome sequencing projects, the rapid accumulation of WGS data, and the clear benefits of pooled multisample variant discovery, *Hydra-Multi* will enable sensitive and accurate SV analysis to be conducted on extremely large datasets using modest computational resources.

## Funding

This work was supported by an NIH/NHGRI [1R01HG006693-01 to A.R.Q.] and an NIH New Innovator Award [DP2OD006493-01] and a Burroughs Wellcome Fund Career Award [to I.M.H.]

*Conflict of interest*: none declared.

## Supplementary Material

Supplementary Data
