# Supplementary material for: Population-based structural variation discovery with Hydra-Multi
Source: Bioinformatics. 2014 Dec 2;31(8):1286–9. doi: 10.1093/bioinformatics/btu771 (PMC4393510; doi:10.1093/bioinformatics/btu771)
Supplement: Supplementary Data [file supp_31_8_1286__index.html]

Population-based structural variation discovery with Hydra-Multi — Population-based structural variation discovery with Hydra-Multi — Population-based structural variation discovery with Hydra-Multi — Supplementary Data 

# Population-based structural variation discovery with Hydra-Multi

## Supplementary Data

files

**Files in this Data Supplement:**

- Supplementary Data - docx file
