# Supplementary material for: Population-based structural variation discovery with Hydra-Multi
Source: Bioinformatics. 2014 Dec 2;31(8):1286–9. doi: 10.1093/bioinformatics/btu771 (PMC4393510; doi:10.1093/bioinformatics/btu771)
Supplement: Supplementary Data [file supp_btu771_resubSupplemental_031114.docx]

| Genome analysis  Population-based structural variation discovery with Hydra-Multi  Michael R. Lindberg^1^, Ira M. Hall^1,2*^ and Aaron R. Quinlan^1,2,3*^  ^1^ Department of Biochemistry and Molecular Genetics, University of Virginia, Charlottesville, VA, USA  ^2^ Department of Public Health Sciences, University of Virginia, Charlottesville, VA, USA.  ^3^ Center for Public Health Genomics, University of Virginia, Charlottesville, VA, USA  Received on XXXXX; revised on XXXXX; accepted on XXXXX  Associate Editor: XXXXXXX |
| --- |

1. Introduction

The following is supplementary information for *Population-based structural variation discovery with Hydra-Multi*, containing supplementary figures, tables, and methods.

2. Supplementary Figures

**Supplementary Figure S1. *Hydra-Multi* workflow.** The algorithm consists of the following steps: configuration, discordant alignment extraction, assembly and sorting, and combining for SV breakpoint finalizing. The extraction of discordant reads, as well as assembly and sorting occur in parallel.


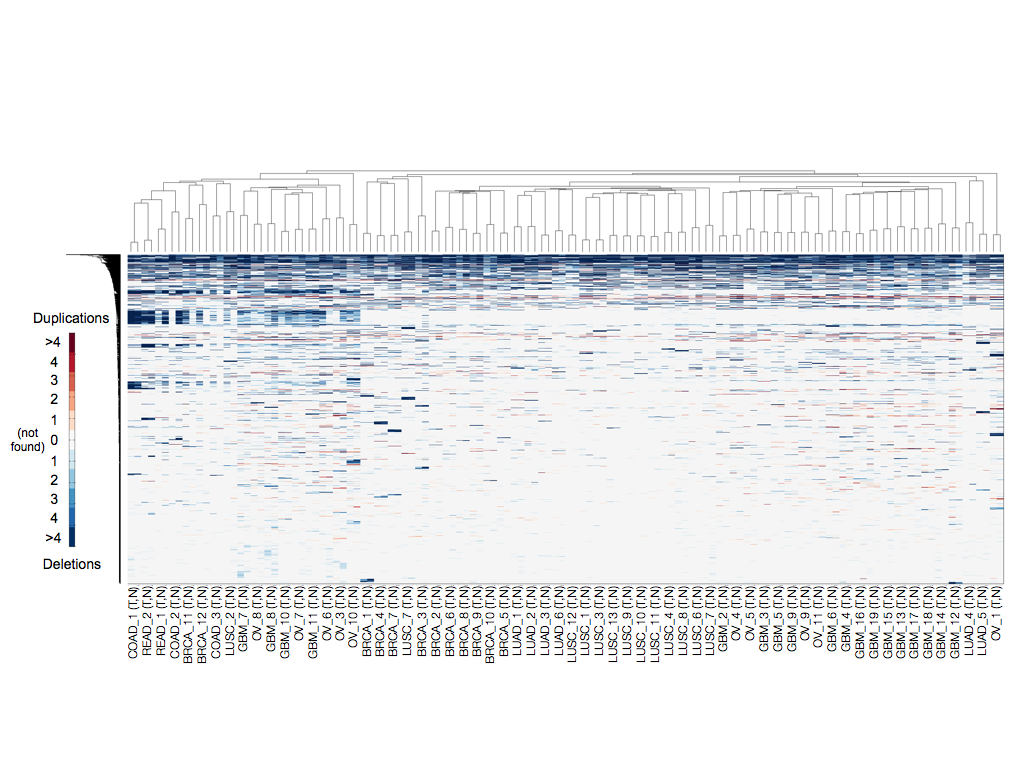


**Supplementary Figure S2. Clustergram of tumor and matched normal germline breakpoints.** Hierarchical clustering of 64 tumor-normal genome pairs from The Cancer Genome Atlas based on 11,994 high-quality germline deletions (blue) and duplications (red), ≤ 1 Megabase in size, made with the Matlab Clustergram function using Spearman correlation distance and Ward linkage. The y-axis is the 11,994 breakpoints, where the number of reads supporting the event is the value, indicated by the intensity of the cell color. Along the X-axis are the samples used in this study: 12 invasive breast cancers (BRCA), 3 colon adenocarcinomas (COAD), 18 glioblastomas (GBM), 6 lung adenocarcinomas (LUAD), 13 lung squamous cell carcinomas (LUSC), 11 ovarian cancers (OV), and 2 renal adenomas (READ) where tumor-normal pairs (denoted by T_N). The tumor-normal pairs are seen to cluster together given their breakpoints, as they are most genetically similar to each other.

**Supplementary Figure S3. Runtimes and speed-up with respect to input size and processor usage with 32 1KGP samples**. Random sequential subsets of 1 to 32, i.e. e.g. n = 1, 2, 4, 8, 16 and 32 dataset(s) were analyzed 3 times to create each of the dataset benchmarks. Runtimes were determined with an increasing number of processors (1:blue, 2:red, 4:green, and 8:black). *Hydra-Multi* was executed using the specified number of processes spawned on each subset and the runtime was measured in minutes. **(A)** The average runtime (minutes) across 3 random samplings at each dataset benchmark subset. Error bars represent 95% confidence intervals. **(B)** All runtimes (minutes) of the random samplings, 3 at each dataset benchmark, plotted against the number of discordant read-pairs (Millions) analyzed and a least-squares regression line.

**Supplementary Table S1. List of TCGA datasets and tissue source**. Datasets appear in consecutive tumor-normal pairs.

| **TCGA Sample Name (n=129)** | **Source Tissue** |
| --- | --- |
| TCGA-A2-A04P-01A-31D-A128-09 | Tumor |
| TCGA-A2-A04P-10A-01D-A128-09 | Blood |
| TCGA-A2-A04T-01A-21D-A128-09 | Tumor |
| TCGA-A2-A04T-10A-01D-A128-09 | Blood |
| TCGA-A2-A0CM-01A-31D-A128-09 | Tumor |
| TCGA-A2-A0CM-10A-01D-A128-09 | Blood |
| TCGA-A2-A0D0-01A-11D-A128-09 | Tumor |
| TCGA-A2-A0D0-10A-01D-A128-09 | Blood |
| TCGA-A2-A0D2-01A-21D-A128-09 | Tumor |
| TCGA-A2-A0D2-10A-01D-A128-09 | Blood |
| TCGA-A7-A0CE-01A-11D-A12L-09 | Tumor |
| TCGA-A7-A0CE-11A-21D-A12L-09 | Normal Tissue |
| TCGA-AO-A0J4-01A-11D-A128-09 | Tumor |
| TCGA-AO-A0J4-10A-01D-A128-09 | Blood |
| TCGA-AO-A0J6-01A-11D-A128-09 | Tumor |
| TCGA-AO-A0J6-10A-01D-A128-09 | Blood |
| TCGA-BH-A0B3-01A-11D-A128-09 | Tumor |
| TCGA-BH-A0B3-11B-21D-A128-09 / TCGA-BH-A0B3-10A-01D-A128-09 | Blood and Normal Tissue |
| TCGA-BH-A0B9-01A-11D-A128-09 | Tumor |
| TCGA-BH-A0B9-10A-01D-A128-09 | Blood |
| TCGA-BH-A0E0-01A-11D-A128-09 | Tumor |
| TCGA-BH-A0E0-10A-01D-A128-09 | Blood |
| TCGA-BH-A0WA-01A-11D-A128-09 | Tumor |
| TCGA-BH-A0WA-10A-01D-A128-09 | Blood |
| TCGA-AA-3518-01A-02D-1525-10 | Tumor |
| TCGA-AA-3518-10A-01D-1525-10 | Blood |
| TCGA-AA-3534-01A-01D-1525-10 | Tumor |
| TCGA-AA-3534-10A-01D-1525-10 | Blood |
| TCGA-AA-A00R-01A-01D-A126-10 | Tumor |
| TCGA-AA-A00R-10A-01D-A126-10 | Blood |
| TCGA-06-0145-01A-01D-0507-08 | Tumor |
| TCGA-06-0145-10A-01D-0507-08 | Blood |
| TCGA-06-0152-01A-02D-0507-08 | Tumor |
| TCGA-06-0152-10A-01D-0507-08 | Blood |
| TCGA-06-0155-01B-01D-0932-09 | Tumor |
| TCGA-06-0155-10A-01D-0703-09 | Blood |

| TCGA-06-0185-01A-01D-0507-08 | Tumor |
| --- | --- |
| TCGA-06-0185-10B-01D-0507-08 | Blood |
| TCGA-06-0188-01A-01D-0373-08 | Tumor |
| TCGA-06-0188-10B-01D-0373-08 | Blood |
| TCGA-06-0208-01A-01D-0512-09 | Tumor |
| TCGA-06-0208-10A-01D-0512-09 | Blood |
| TCGA-06-0214-01A-02D-0512-09 | Tumor |
| TCGA-06-0214-10A-01D-0512-09 | Blood |
| TCGA-06-0648-01A-01D-0507-08 | Tumor |
| TCGA-06-0648-10A-01D-0507-08 | Blood |
| TCGA-06-0877-01A-01D-0512-09 | Tumor |
| TCGA-06-0877-10A-01D-0512-09 | Blood |
| TCGA-06-0881-01A-02D-0512-09 | Tumor |
| TCGA-06-0881-10A-01D-0512-09 | Blood |
| TCGA-06-1086-01A-02D-0932-09 | Tumor |
| TCGA-06-1086-10A-01D-0703-09 | Blood |
| TCGA-14-0786-01B-01D-0932-09 | Tumor |
| TCGA-14-0786-10A-01D-0703-09 | Blood |
| TCGA-14-1401-01A-01D-0703-09 | Tumor |
| TCGA-14-1401-10A-01D-0703-09 | Blood |
| TCGA-14-1454-01A-01D-0932-09 | Tumor |
| TCGA-14-1454-10A-01D-0703-09 | Blood |
| TCGA-14-1459-01A-01D-0932-09 | Tumor |
| TCGA-14-1459-10A-01D-0703-09 | Blood |
| TCGA-16-1063-01B-01D-0932-09 | Tumor |
| TCGA-16-1063-10A-01D-0703-09 | Blood |
| TCGA-16-1460-01A-01D-0932-09 | Tumor |
| TCGA-16-1460-10A-01D-0703-09 | Blood |
| TCGA-26-1438-01A-01D-0932-09 | Tumor |
| TCGA-26-1438-10A-01D-0703-09 | Blood |
| G7164.TCGA-44-2665-01A-01D-0969-08.4 | Tumor |
| G7164.TCGA-44-2665-10A-01D-0969-08.3 | Blood |
| G7165.TCGA-44-2666-01A-01D-0969-08.7 | Tumor |
| G7165.TCGA-44-2666-10A-01D-0969-08.8 | Blood |
| G7166.TCGA-55-1594-01A-01D-0969-08.6 | Tumor |
| G7166.TCGA-55-1594-11A-01D-0969-08.7 | Normal Tissue |
| G7167.TCGA-55-1596-01A-01D-0969-08.6 | Tumor |
| G7167.TCGA-55-1596-11A-01D-0969-08.8 | Normal Tissue |
| G7168.TCGA-67-3771-01A-01D-0969-08.7 | Tumor |
| G7168.TCGA-67-3771-10A-01D-0969-08.9 | Blood |

| G7169.TCGA-67-3772-01A-01D-0969-08.6 | Tumor |
| --- | --- |
| G7169.TCGA-67-3772-10A-01D-0969-08.7 | Blood |
| G12518.TCGA-34-2596-01A-01D-0963-08.1 | Tumor |
| G12518.TCGA-34-2596-11A-01D-0963-08.1 | Normal Tissue |
| G12519.TCGA-34-2600-01A-01D-0963-08.2 | Tumor |
| G12519.TCGA-34-2600-11A-01D-0963-08.1 | Normal Tissue |
| G12520.TCGA-60-2711-01A-01D-0963-08.1 | Tumor |
| G12520.TCGA-60-2711-11A-01D-0963-08.1 | Normal Tissue |
| G13226.TCGA-21-1078-01A-01D-1521-08.1 | Tumor |
| G13226.TCGA-21-1078-11A-01D-1521-08.1 | Normal Tissue |
| G13227.TCGA-43-3394-01A-01D-1553-08.1 | Tumor |
| G13227.TCGA-43-3394-11A-01D-1553-08.1 | Normal Tissue |
| G13229.TCGA-56-1622-01A-01D-1521-08.1 | Tumor |
| G13229.TCGA-56-1622-11A-01D-1521-08.3 | Normal Tissue |
| G13230.TCGA-60-2713-01A-01D-1522-08.1 | Tumor |
| G13230.TCGA-60-2713-11A-01D-1522-08.3 | Normal Tissue |
| G13231.TCGA-60-2724-01A-01D-1522-08.2 | Tumor |
| G13231.TCGA-60-2724-11A-01D-1522-08.2 | Normal Tissue |
| G8889.TCGA-60-2695-01A-01D-1180-08.1 | Tumor |
| G8889.TCGA-60-2695-11A-01D-1180-08.1 | Normal Tissue |
| G8890.TCGA-60-2722-01A-01D-1180-08.2 | Tumor |
| G8890.TCGA-60-2722-11A-01D-1180-08.1 | Normal Tissue |
| G8891.TCGA-66-2756-01A-01D-1180-08.1 | Tumor |
| G8891.TCGA-66-2756-11A-01D-1180-08.1 | Normal Tissue |
| G8892.TCGA-66-2757-01A-01D-1180-08.1 | Tumor |
| G8892.TCGA-66-2757-11A-01D-1180-08.1 | Normal Tissue |
| G8893.TCGA-66-2766-01A-01D-1180-08.2 | Tumor |
| G8893.TCGA-66-2766-11A-01D-1180-08.1 | Normal Tissue |
| TCGA-04-1371-01A-01D-0516-08 | Tumor |
| TCGA-04-1371-11A-01D-0516-08 | Normal Tissue |
| TCGA-13-0723-01A-02D-0447-09 | Tumor |
| TCGA-13-0723-10B-01D-0447-09 | Blood |
| TCGA-13-0725-01A-01D-0465-08 | Tumor |
| TCGA-13-0725-10B-01D-0446-08 | Blood |
| TCGA-13-0751-01A-01D-0446-08 | Tumor |
| TCGA-13-0751-10A-01D-0446-08 | Blood |
| TCGA-13-0890-01A-01D-0421-09 | Tumor |
| TCGA-13-0890-10A-01D-0421-09 | Blood |
| TCGA-13-1411-01A-01D-0515-09 | Tumor |
| TCGA-13-1411-10A-01D-0515-09 | Blood |

| TCGA-24-0980-01A-01D-0515-09 | Tumor |
| --- | --- |
| TCGA-24-0980-10A-01D-0515-09 | Blood |
| TCGA-24-0982-01A-01D-0516-08 | Tumor |
| TCGA-24-0982-10C-01D-0516-08 | Blood |
| TCGA-24-1103-01A-01D-0515-09 | Tumor |
| TCGA-24-1103-10A-01D-0515-09 | Blood |
| TCGA-25-1319-01A-01D-0516-08 | Tumor |
| TCGA-25-1319-10A-01D-0516-08 | Blood |
| TCGA-AF-2689-01A-01D-1525-10 | Tumor |
| TCGA-AF-2689-10A-01D-1525-10 | Blood |
| TCGA-AG-3593-01A-01D-1525-10 | Tumor |
| TCGA-AG-3593-10A-01D-1525-10 | Blood |

**Supplementary Table S2. List of 1KGP datasets and population of origin**. Datasets appear in their respective population source.

| **1KGP Sample Name (n=65)** | **Source population** |
| --- | --- |
| NA12878 (5x and 50x) | CEU |
| NA06989 | CEU |
| NA12272 | CEU |
| NA18626 | CHB |
| HG00530 | CHS |
| HG00533 | CHS |
| HG00534 | CHS |
| HG00565 | CHS |
| HG00566 | CHS |
| HG00581 | CHS |
| HG00584 | CHS |
| HG00592 | CHS |
| HG00596 | CHS |
| HG00657 | CHS |
| HG00671 | CHS |
| HG00672 | CHS |
| HG00684 | CHS |
| HG00690 | CHS |
| HG00693 | CHS |
| HG00698 | CHS |
| HG00704 | CHS |
| HG00173 | FIN |
| HG00177 | FIN |
| HG00180 | FIN |
| HG00183 | FIN |
| HG00185 | FIN |
| HG00188 | FIN |
| HG00272 | FIN |
| HG00275 | FIN |
| HG00276 | FIN |
| HG00278 | FIN |
| HG00280 | FIN |
| HG00281 | FIN |
| HG00282 | FIN |
| HG00284 | FIN |
| HG00285 | FIN |
| HG00306 | FIN |
| HG00310 | FIN |
| HG00324 | FIN |
| HG00326 | FIN |
| HG00329 | FIN |
| HG00334 | FIN |
| HG00357 | FIN |
| HG00366 | FIN |
| HG00369 | FIN |
| HG00112 | GBR |
| HG00120 | GBR |
| HG00126 | GBR |
| HG00142 | GBR |
| HG00143 | GBR |
| HG00154 | GBR |
| HG00244 | GBR |
| HG00246 | GBR |
| NA19081 | JPT |
| NA19082 | JPT |
| NA19084 | JPT |
| NA19428 | LWK |
| NA19468 | LWK |
| HG00551 | PUR |
| HG00554 | PUR |
| HG00638 | PUR |
| HG00641 | PUR |
| HG00740 | PUR |
| HG01107 | PUR |
| HG01108 | PUR |

3. Supplementary Methods

*Data processing and implementation.* The data was obtained from TCGA and 1KGP available on dpGAP and the EBI/NCBI FTPs. The datasets used are listed in Supplementary Tables S1 and S2, for TCGA and 1KGP respectively. The TCGA datasets were processed slightly different than the standard *Hydra-Multi* analysis; a detailed explanation of how this was done can be found in Malhotra *et al*.. This approach assumes that bam files may contain multiple sequencing libraries. Under this assumption, the sample statistics that are used to determine which read-pairs are proper pairs will be more accurate because they are evaluated by library. This is accomplished by several adjustments to parameters and changes to the overall workflow compared to the typical use of *Hydra-Multi*. The 1KGP datasets were analyzed using the more standard, user-friendly implementation, as described on the website: <https://github.com/arq5x/Hydra>, which maintains that bam files contain a single sequencing library.

*1KGP accuracy performance analyses.* From the 1KGP datasets, the NA12878 dataset (50x coverage from EBI) of the CEU family cohort and 64 random bam files (~5x coverage from NCBI, Supplementary Table S2) were used for performance analyses. The 50x NA12878 dataset was randomly subsampled by read-pairs to roughly 5x coverage, simulating a comparable 5x low coverage dataset for this individual. The 64 datasets were of approximately similar size, between 12 and 27 Gb, to prevent large differences in coverage from dramatically changing runtimes or SV support. A necessary step was the realignment of both NA12878 datasets (5x and 50x) using *BWA* (Li and Durbin, 2009) and duplicate removal with *SAMBLASTER* (Faust and Hall, 2014) with default parameters. Realignment of these datasets was necessary due to the different reference genome versions used for original alignment of 1KGP and Illumina Platinum datasets. These datasets were then used to compare the relative performance of *Hydra-Multi, GASVPro* v2.0 (Sindi. *et al.,* 2009), and an unpublished multi-sample version of *DELLY* v0.5.3 *(*Rausch *et al.,* 2012, <https://github.com/tobiasrausch/delly>). In these comparisons, the number of true positives and false positives were measured for each tool in three scenarios: 1) analyzing NA12878 at 5x coverage by itself, 2) NA12878 at 50x coverage by itself, and finally 3) NA12878 (5x) with the 64 random datasets. To measure the true and false positive rates, we used a truth set consisting of 3,077 non-overlapping validated deletions in NA12878 from the Mills *et al.* study.

Putative calls from *Hydra-Multi*, *GASVPro*, and *DELLY* were generated in each of the analyses using similar parameters for each tool. In all analyses, a minimum mapping quality of 20 was required for each read. The input deviation parameter values across all tools were made to be equivalent. As such, the deviation parameters for *Hydra-Multi* and *DELLY* were set to 5 and 8 MADs for each of the respective analyses. The corresponding computed *Hydra-Multi* values at these settings (5 and 8 median absolute deviations or MADs) were used as the input *GASVPro’s “*LminLxmax” values. This was necessary because *GASVPro* calculates fragment size distributions using standard deviations from the input parameter, while the other two tools use MADs. Standard deviations can be significantly larger than MADs, making the comparison incongruous. The tool specific "punt" parameter for *Hydra-Multi* was set to a value corresponding to 5 times the summed mean dataset coverage (e.g., 25 for one 5x dataset, or 250 for ten 5x datasets). At the outset of variant calling, a minimum support of two read-pairs (read-pairs and/or split-reads for *DELLY*) was required for discovery. Because all three tools report breakpoints in three different output formats, the outputs for all tools were converted into BEDPE format using zero-based, half-open arithmetic to make a fair comparison. The *GASVPro* regions file reports the breakpoint boundary points of the final polygons constructed. The midpoint the left and right boundaries were calculated and padded with 100 bp in both directions to create a set of two 200 bp intervals. Next, the single base start and end coordinates reported by *DELLY* in VCF format were given 100 bp of slop in both directions to make two intervals of 200 bp. The final reported *Hydra-Multi* breakpoint footprints were made into breakpoint intervals by drawing two 200 bp intervals inward from the read-mapping "footprints", i.e. end1 + 200 bp and start2 – 200bp. In each conversion to BEDPE intervals, the number supporting read-pairs were tracked; however, this was not possible in the analysis using 64 datasets and NA12878 at 5x. First, *GASVPro* is not currently a multi-sample caller and therefore could not be evaluated in a multi-sample analysis. For *DELLY*, the presence or absence of a call in NA12878 from the multi-sample analysis was assessed either by the reported genotype (GT) or whether NA12878 contributed at least one high-quality variant pair (DV) to the breakpoint. GT is equal to DV for a single sample analysis requiring support of 4 – 10 read pairs, thus only GT was reported in Figure 1A and 1B, For *Hydra-Multi*, a call was considered present in NA12878 when at least one original read-pair from NA12878 was used in making the final breakpoint call. Since both *DELLY* and *Hydra-Multi* report the total read support across all datasets, this was used as a filtering criterion in the ROC curves.

All putative breakpoint interval sets and the truth set were filtered to remove GL, MT, and Y chromosome calls, as well as any interval that overlapped with a set of exclude regions (<https://github.com/cc2qe/speedseq/blob/master/annotations/ceph18.b37.lumpy.exclude.2014-01-15.bed>). These exclude regions are comprised of very high sequence coverage in the 17-member CEPH 1463 pedigree. The read-depth at these locations was greater than 2*mode + 3 standard deviations, as found by aligning with *BWA MEM* (Li, 2013) and measuring the depth with *BEDTools* (Quinlan and Hall, 2010), whereby the autosomal and sex chromosomes were analyzed separately. The filtered call sets from each of the three tools (both 5 and 8 MADs) were evaluated at varying levels of total support (4 to 10 read-pairs) in three scenarios. True positive were calculated by finding the number of uniquely identified truth set deletions. An identified truth set deletion was one with which the two truth set breakpoint intervals intersected with any pair of breakpoint intervals reported in a given comparison. A false positive was defined as the number of SVs reported by a tool that did not identify a truth set deletion.

*TCGA tumor-normal false discovery rate estimations.* Given c = n choose k, false discovery rates were calculated by using combinations of the total number of tumor-normal pairs, n, as more pairs, k, are considered across the set of high-confidence breakpoints found in the TCGA datasets (Supplemental Table S1). For practical purposes, c was limited to 1000 random combinations due to it being very large for most values k. Therefore, c = 64 choose k for k = 1 to 64, where c ≤ 1000, which limits the number of combinations explored to 1000. Thus, for each c, false discoveries were determined by the presence or absence of somatic and germline in all breakpoints. A breakpoint is said to be a false discovery for any c if it is seen in a normal sample genome of any one tumor-normal pair and it is not in it’s matched tumor sample or another tumor-normal pair. Each breakpoint was tested to be a false discovery in all combinations c and the false discovery rate at each value k was calculated. The false discovery rate was the ratio of the total private normal sample breakpoints to the total private tumor sample breakpoints in each k.

*1KGP speed and scalability performance analyses*. Maximum memory and runtimes were determined with the runit utility (<https://github.com/lh3/misc/tree/master/sys/runit>). The three previously described deletion detection scenarios were included in the speed and scalability performance analyses. The maximum memory usage and total runtime for the three scenarios with 8 MADs were recorded. An additional analysis to simulate a large number of input datasets for *Hydra-Multi* and *DELLY* consisted of repeating instances of the NA12878 datasets. In all speed and scalability measurements, *Hydra-Multi* was allocated 8 processors, *DELLY* was permitted up to 32 threads, and *GASVPro* was given 20 Gb for the Java Virtual Machine. *Hydra-Multi’*s punt parameter was also adjusted to 5 times the summed mean input dataset coverage. Runtime measurement of each processor usage versus the number of datasets as well as discordant read-pairs was done using 1KGP datasets. The previous 64 datasets were sequentially subsampled at random 3 times, e.g. n = 1, 2, 4, 8, 16, and 32, for each benchmark.

*Hardware specifications and utilities*. All analyses were done using a single compute node running CentOS 2.6.32-358.2.1.el6.x86_64 on 16 Intel Xeon E5-2670 CPUs with 128 Gb random access memory and an array of twelve 4 Tb hard disks spinning at 7200 rpm*.* All interval intersections were done with *BEDTools* (Quinlan and Hall, 2010).

4. Hydra-Multi Algorithm

*Set up and configuration.* A configuration file is necessary to provide a unique identifier and file path defining the appropriate alignment file for each DNA library (or sample) interrogated for SV breakpoints. Before running *Hydra-Multi*, it is imperative that these bam files have duplicate reads (molecules) removed. The configuration file must define i) a central tendency statistic for the insert size for the library – *mean* or *median* are recommended, ii) a standardized measure of the variance in the library – typically the *standard deviation* or *median absolute deviation*, and iii) the number of units of variance (e.g., “6” for six standard deviations) that should be used to define a proper pair or *concordant* alignment when pairs align in the +,- (forward, reverse) orientation within expected genomic space.

*Routing discordant alignments by genomic coordinates and strand.* The algorithm begins by routing similar *discordant* alignments, i.e., paired-end mappings with either an unexpected insert size (greater than the central statistic + number of variance metrics) or an aberrant strand combination (-,+;+,+;-,- orientations), from all libraries (or samples) defined in the configuration file to common alignment files using the hydra-router tool. In effect, this step consolidates all mappings from each of the libraries (or samples) into discrete files that are likely to support the same breakpoints. For example, all paired-end mappings that indicate deletions on chromosome 10 would be place in a file called *chr10.chr10.+.-*. The benefit of this routing step is that each discrete file of discordant mappings can be processed independently, since the mappings therein can only support specific types of SV breakpoints involving the defined chromosomes. In turn, this independence facilitates a high degree of parallelism when screening for candidate breakpoints.

*Identifying candidate breakpoint “clusters”.* Each routed file of discordant alignments is then sorted by each mapping’s “leftmost” start position (that is, the position with the lowest start coordinate). Sorting in this manner organizes the mappings from each library (or sample) such that alignments supporting the same breakpoint are aggregated. In order to maximize sorting efficiency, we developed a custom C++ implementation (<https://github.com/arq5x/kway-mergesort>) of the k-way, memory assisted merge-sort algorithm. Importantly, as sorting the discordant mappings is one of the more computationally intensive steps of our approach, this approach combines the benefits of a disk-based merge-sort algorithm with the speed of in-memory sorting. This allows the user to define precisely how much memory should be allocated to the sorting step and avoids prohibitive memory consumption while providing fast sorting times.

*Greedy breakpoint reconstruction.* After sorting mappings based on their start position, it is common for mappings supporting different rearrangement events to be sorted together, especially when integrating data from hundreds of samples and/or from highly rearranged genomes. *Hydra-Multi* addresses this in two phases. First, it scans each sorted file to build clusters with mappings that have the potential to support the same breakpoint based on the supplied variance statistics. A cluster is terminated once an a mapping is encountered whose start coordinate is to the “right” of the current cluster’s rightmost end coordinate; by definition, such a mapping cannot support the same breakpoint as the mappings already in the cluster. The clusters are then sorted by relative support and assembled into “contigs” which represents a larger interval of assembled space in the genome. Once assembled, the contributing cluster is removed from the pool of usable clusters that can corroborate subsequent breakpoints in further assemblies. A cluster may also be terminated in regions of overly complex genomic rearrangements. These regions can cause excessive runtimes and may be averted by “punting” once a certain number of mappings have been attributed to a putative cluster. A reasonable heuristic for this parameter is 5 multiplied by the summed mean coverage of the datasets to be analyzed (e.g., 250 for ten 5x genomes).

*Punt parameter.* The purpose of "punting" is to avoid needless increases in runtimes caused by the unnecessary analysis of genomic regions that have an abnormally high concentration of discordant read pairs. The vast majority of loci with this signature are known read alignment artifacts stemming from mis- or unassembled regions of the reference genome. These loci are typically excluded from downstream analyses; to improve runtimes, we exclude them before breakpoint detection. Under the provided usage parameters, this strategy has essentially zero effect on the sensitivity of detecting germline breakpoints. However, it is important to note that under certain usage scenarios – for example, analysis of a single tumor/normal pair – the decision to "punt" may cause variants to be missed within highly amplified genomic segments. As a reasonable punt parameter, we recommend 5 times the summed mean coverage of all datasets included in an analysis. For example, in an analysis of 10 cancer genomes sequenced to 50x coverage, the recommended punt parameter would be 2500. This setting should be able to detect an average (depending on ploidy) of about 10 copies per genome or roughly 100 copies in total. Thus, if a locus is amplified to extremely high levels (>100 copies) in one genome, or at moderate levels (~10 copies) across most or all genomes included in an analysis, it is possible that breakpoints will be missed by *Hydra-Multi* using the recommended punt parameter. However, this should not be a major concern for the following reasons. First, even if such loci are not excluded, in practice it is generally quite difficult to interpret breakpoint calls within amplified genomic regions, and thus most studies do not attempt breakpoint analysis at such loci. Second, and under most experimental designs the relevant gene amplification will be detected by read-depth analysis, which is much more suited for this goal. Third, for analyses where it is necessary to include high depth genomic regions, the user can input arbitrarily high punt parameter values to ensure that no breakpoints are missed (at the expense of increased runtimes).

5 References

Faust, G. F. and Hall, I.M. (2014). SAMBLASTER: fast duplicate marking and structural variant read extraction. *Bioinformatics*, 30, 2303-2305.

Li, H. and Durbin, R. (2009) Fast and accurate short read alignment with Burrows-Wheeler transform. *Bioinformatics*, 25, 1754-1760.

Li, H. (2013) Aligning sequence reads, clone sequences, and assembly contigs with BWA-MEM. *arXiv*, 1303.3997, (http://arxiv.org/pdf/1303.3997v2.pdf)

Malhotra, A. *et al.* (2013) Breakpoint profiling of 64 cancer genomes reveals numerous complex rearrangements spawned by homology-independent mechanisms. Ge*nome Research*, 23, 762-776.

Mills, R. E. *et al.* (2011) Mapping copy number variation by population-scale genome sequencing. *Nature* **470**, 59-65.

Quinlan, A. R. and Hall I.M. (2010) BEDTools: a flexible suite of utilities for comparing genomic features. *Bioinformatics* **26**, 841-842.

Rausch, T. *et al.* (2012) DELLY: structural variant discovery by integrated paired-end and split-read analysis. *Bioinformatics,* **28, i333-i339.**

Sindi, S. *et al.* (2009) A geometric approach for classification and comparison of structural variants. *Bioinformatics*, **25**, 222-230.
